# Tcf4 regulates dendritic spine density and morphology in the adult brain

**DOI:** 10.1371/journal.pone.0199359

**Published:** 2018-06-22

**Authors:** Sophie Crux, Jochen Herms, Mario M. Dorostkar

**Affiliations:** 1 German Center for Neurodegenerative Diseases (DZNE), Munich, Germany; 2 Munich Cluster of Systems Neurology (SyNergy), Ludwig–Maximilian–University, Munich, Germany; 3 Center for Neuropathology, Ludwig–Maximilian–University, Munich, Germany; University of Victoria, CANADA

## Abstract

Tcf4 is a transcription factor which regulates neurogenesis and neuronal migration in the brain. In humans, loss of function of Tcf4 leads to the rare neurodevelopmental disorder Pitt-Hopkins syndrome, which is characterized by intellectual disability, developmental delay and autistic behavior. We analyzed the consequences of functional loss of Tcf4 on dendritic spines in mature principal neurons. To this end, we crossed mice in which the DNA-binding domain of the *Tcf4* gene is flanked by LoxP sites to mice expressing tamoxifen-inducible cre recombinase in a sparse subset of fluorescently labelled neurons (SlickV line). This resulted in a mouse model with an inducible functional knockout of Tcf4 in a subset of cortical and hippocampal neurons, in which we analyzed dendritic spines, which are the morphological correlate of excitatory postsynapses. Heterozygous as well as homozygous loss of Tcf4 led to a reduction in the number of dendritic spines in the cortex as well as in the hippocampus. This was accompanied by morphological changes of dendritic spines. These results suggest that Tcf4 is involved in synaptic plasticity in mature neurons, and functional loss of Tcf4 may contribute to the neurological symptoms in Pitt-Hopkins syndrome.

## Introduction

Tcf4 belongs to the basic-helix-loop-helix (bHLH) family of proteins, which act as transcription factors. In the developing nervous system bHLH proteins regulate neurogenesis, and migration of postmitotic neurons: Initially, cycling progenitor cells are committed to a neuronal fate through activation of Notch signalling [1], while later proper localization of neurons is established [2]. In the brain Tcf4 interacts with the class II bHLH transcription factors Math1, HASH1, and neuroD2 [3]. Binding of the calcium sensor calmodulin to Tcf4 inhibits transcriptional activation through interaction with the DNA binding domain [3].

Mutations or deletions of the human *Tcf4* gene cause Pitt-Hopkins syndrome, a rare developmental disorder which is characterized by severe intellectual disability, developmental delay and autistic behavior [4]. In mice, constitutive *Tcf4* knockout disrupts normal brain development [2]. In contrast, a gain of function in *Tcf4* is associated with a higher risk in developing schizophrenia [3, 5].

A number of signaling cascades involved in brain development have been shown to play important roles in synaptic function in adults. For instance, several proteins involved WNT signaling, which plays important roles in cortical development [6–8], were shown to modulate synaptic function in adults [9–12]. Similarly, proteins involved in CREB signaling, which play a role in cerebellar development [13] affect synaptic transmission [14–16]. All three signaling cascades (TCF4, CREB, WNT) have in common that they have been identified to mediate the risk for schizophrenia in humans [3, 5, 17–19], thus highlighting a possible role in the maintenance of synaptic function independently of their developmental functions. However, the synaptic function of Tcf4 in the adult brain has not yet been examined.

In order to study the effects of Tcf4 on the regulation of adult synapses, we generated mice with inducible knockout of Tcf4 in a sparse subset of fluorescently labelled neurons. These permit visualization of dendritic spines to determine alterations in their number or shape [12, 20]. Functionally, dendritic spines are the morphological correlates of excitatory postsynapses, where specialized synaptic proteins, such as scaffolding proteins and ion channels are clustered [21, 22]. The structural shape of spines typically reflects specific functional properties. For instance, increases in spine head size help accommodate larger numbers of neurotransmitter receptors, while shortening and widening of spine necks decreases the electrical resistance of the spine neck, thereby leading to larger excitatory postsynaptic potentials [23]. Thus, at dendritic spines neuronal information is received and integrated and the numbers and shapes of spines reflect biological function [24].

Dendritic spines are typically classified based on their morphology into three groups [25–27]: Mushroom spines possess a large head and thin, clearly discernible neck. Stubby spines similarly possess a large head but lack a discernible neck. Thin spines are long and slender and possess a smaller head than the previous two classes. These different shapes are thought to cause functional differences. For example, mushroom and stubby spines with their large heads, which provide a larger area for neurotransmitter receptors, are thought to be more stable than thin spines, which are thought to be more plastic [25–27].

## Materials & methods

### Transgenic mice

The animal research protocols were approved by the animal welfare committee of the Ludwig-Maximilian-University Munich and the government of Upper Bavaria (Ref. Nr. 55.2-1-54-2532-62-12).

Animals were sacrificed by transcardiac perfusion with phosphate-buffered saline (PBS) followed by PBS containing 4% PFA (w/v) in deep ketamine/xylazine anesthesia.

SlickV mice, which coexpress a drug-inducible form of cre recombinase and the fluorescent protein YFP in a subset of neurons [28], were crossed with floxed *Tcf4* mice, in which of a 4.3 kb fragment of the *Tcf4* gene containing the bHLH exon and 3’ exons, which are responsible for DNA binding and dimerization, is flanked by LoxP sites [29]. In the resultant SlickV×*Tcf4*^*LoxP/LoxP*^ mice, functional knockout of Tcf4 can be induced in a fluorescently labelled subset of neurons in the hippocampus and cortex upon tamoxifen administration. All geneotypes were treated with tamoxifen, applied by oral gavage (0.25 mg/g, tamoxifen dissolved in 95% corn oil and 5% ethanol) once per day for five consecutive days at 10–11 weeks of age followed by transcardiac perfusion 2 weeks after the treatment had concluded.

The mice were bred for research purposes in the animal facility of the Center for Neuropathology, Ludwig—Maximilians—University, Munich, Germany.

Animals were group housed under pathogen-free conditions. All mice were kept at a 12/12 hr light/dark cycle with ad libitum access to food and water.

### Immunohistochemistry

Animals were sacrificed by transcardiac perfusion with phosphate-buffered saline (PBS) followed by PBS containing 4% PFA (w/v) in deep ketamine/xylazine anesthesia. The brains were removed and postfixed in PBS containing 4% PFA over night before cutting 50 μm thick coronal sections on a vibratome (VT 1000S from Leica, Wetzlar, Germany).

Floating sections were permeabilized with 2% Triton X-100 in PBS over night at room temperature, washed 3×10 min with PBS, followed by blocking with 3% Iblock in 0.1% Triton X-100 in PBS for 2h at room temperature. Sections were incubated over night at 4°C with 1:500 anti GFP Alexa 488 antibodies (A21311, Thermofisher) in PBS with 2% Triton-X 100. Sections were finally washed 5×10 min with PBS before mounting them on glass coverslips with Vectashield Hard Set fluorescence conserving media (Vector Laboratories).

### Image acquisition and analysis

Images were acquired on a Zeiss LSM 780, using a 40× oil immersion objective. Figures show maximum intensity projected images, while the analysis was performed in 3D. Spines were counted in z-stacks by manually scrolling through the images. Spine densities refer to the number of spines per dendrite length from which they protrude.

Because of the sparse labelling, dendrites from layer V pyramidal neurons in the somatosensory cortex, ranging from bregma -1.755 to -2.255 mm were considered. Dendrites from CA1 neurons were imaged in the dorsal hippocampus at the same coordinates.

Spine number and morphology was determined by manually tracing dendrites in ZEN software using drawing tools provided in the software and classified by eye based on morphological criteria [30], followed by data extraction using the SpineMiner software [31]. All spinal protrusions from the dendrite counted towards the total number of spines. However, morphological analyses were made on the three main spine classes (mushroom, stubby thin), while filopodia were too rare to be considered for separate statistical analysis.

Morphology illustrations were made with IMARIS (Bitplane, Zürich, Switzerland).

The overview picture was taken with a Zeiss Axio Imager 2 fluorescence microcope with Apotome.

### Statistics

From each animal ten dendrites of approximately 35 μm length from each brain region were analyzed to calculate dendritic spine densities and morphologies. The dendrites belonged to 3–5 neurons, so that, typically 2–4 dendritic segments per neuron were analyzed.

The results of each animal were averaged, and statistical tests were performed on the means of the means, so that N corresponds to animals. A total of six animals per genotype were analyzed. Statistical analyses were performed in Prism 5.04 (GraphPad, La Jolla, CA, USA). When results are stated, mean ± standard error of the mean is given. For multiple comparisons, ANOVA with Tukey’s post hoc test was used. Data were assumed to be normally distributed as, according to the central limit theorem, averages of averages tend towards a normal distribution. This was confirmed empirically using the Shapiro-Wilk normality test.

## Results

**Fig 1:**
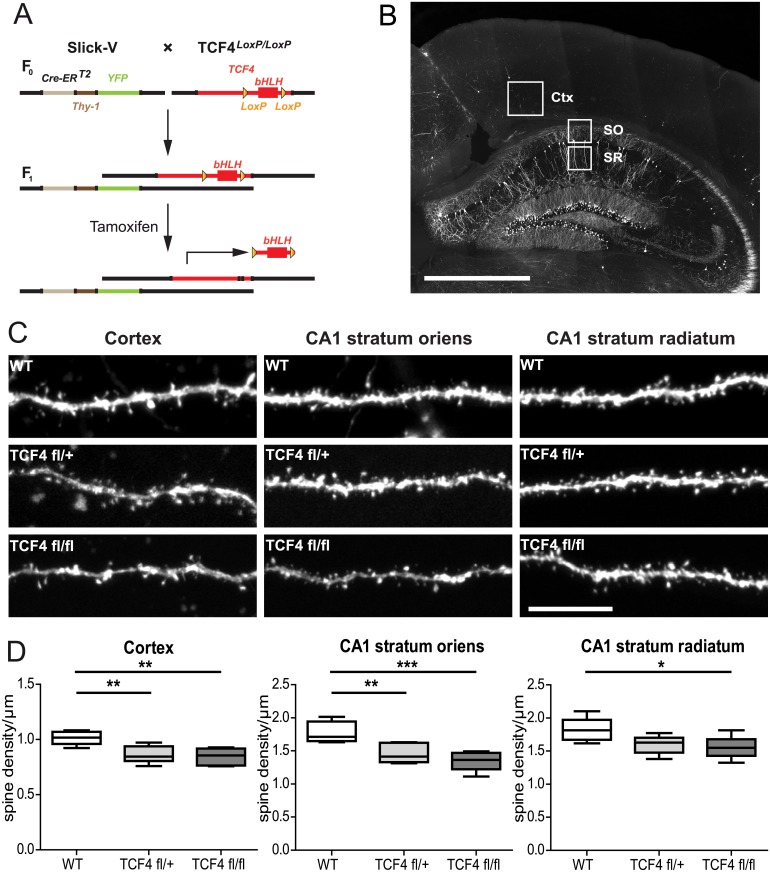
Heterozygous and homozygous loss of TCF4 leads to dendritic spine density decrease in the adult brain. (A) Breeding scheme to obtain inducible TCF4 knockout mice. SlickV mice co-express tamoxifen-inducible cre recombinase (CreERT2) with YFP under the *Thy1* promoter. These animals were crossed with *Tcf4*^*LoxP/LoxP*^ mice, in which a gene fragment containing the bHLH sequence is flanked by LoxP sites. Administration of tamoxifen irreversibly removes part of the *Tcf4* sequence from YFP-expressing neurons resulting in a functional knock out. (B) Coronal section of the brain of a SlickV mouse, YFP stain. Note the sparse labelling in the cortex and CA1 region. Dendritic spines were quantified in the whole cortex (Ctx; the box highlights a YFP expressing neuron in the primary somatosensory area, trunk, layer V) and the CA1 area of the hippocampus in stratum oriens (SO) and stratum radiatum (SR). Scale bar, 1000 μm. (C) Images of dendrites in the cortex, CA1 stratum oriens and CA1 stratum radiatum of WT, *Tcf4*^*fl/+*^ and *Tcf4*^*fl/fl*^ animals. Scale bar, 10 μm. (D) After tamoxifen administration *Tcf4*^*fl/+*^ and *Tcf4*^*fl/fl*^ display decreased spine density in the cortex (left) and in stratum oriens of CA1 (middle) compared to WT; n = 6 mice; ** p < 0.01; ***p < 0.001. In stratum radiatum of CA1 (right) *Tcf4*^*fl/fl*^ display decreased spine density compared to WT; n = 6 mice *p < 0.05. P values are based on ANOVA with Tukey’s multiple comparison test.

In order to study dendritic spines in Tcf4 deficient mice, we crossed mice co-expressing tamoxifen-inducible cre recombinase with YFP in a sparse subset of neurons under the *Thy1* promoter (Slick-V line) [28] with *Tcf4*^*fl/fl*^ mice (Fig 1A) [29], enabling us to visualize dendritic spines of cortical and hippocampal pyramidal cells (Fig 1B). Tcf4 knockout was induced by tamoxifen administration to 10–11 weeks old animals for 5 days, resulting in the deletion of a 4.3 kb fragment of the *Tcf4* gene containing the bHLH exon and 3’ exons, which are responsible for DNA binding and dimerization [29].

First, we quantified the number of dendritic spines of basal dendrites in YFP-labelled layer V cortical neurons (Fig 1C): Dendrites of tamoxifen-treated wildtype controls showed average spine densities of 1.01±0.02/μm (n = 6 mice). Loss of a single allele of *Tcf4* (*Tcf4*^fl/+^) caused a significant reduction in spine density to 0.86±0.03/μm (n = 6 mice; p < 0.01, ANOVA with Tukey’s multiple comparison test). Homozygous loss of *Tcf4* (*Tcf4*^fl/fl^) caused a similar reduction in dendritic spines, to 0.85±0.03/μm (n = 6 mice; p < 0.01 vs. control, ANOVA with Tukey’s multiple comparison test; Fig 1D). We found similar results in CA1 hippocampal neurons: In stratum oriens dendrites wildtype controls showed average spine densities of 1.77±0.07/μm (n = 6 mice), which were reduced to 1.45±0.06/μm in *Tcf4* heterozygous animals (n = 6 mice; p < 0.01, ANOVA with Tukey’s multiple comparison test) and to 1.34±0.06/μm in homozygous *Tcf4* knockout (n = 6 mice; p < 0.001 vs. control, ANOVA with Tukey’s multiple comparison test; Fig 1D). In stratum radiatum dendritic spine density was reduced from 1.83±0.07/μm in wildtype (n = 6 mice) to 1.6±0.06/μm (n = 6 mice) in heterozygous and to 1.56±0.07/μm in homozygous Tcf4 knockout (n = 6; p < 0.05 vs. control, ANOVA with Tukey’s multiple comparison test; Fig 1D).

**Fig 2:**
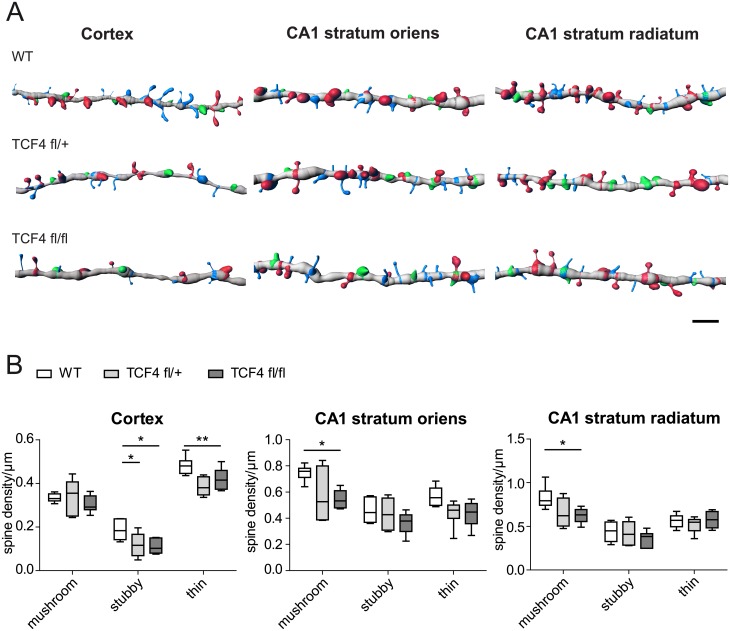
Heterozygous and homozygous loss of TCF4 affects the dendritic spine morphology in the adult brain. (A) Illustrative reconstructions of dendritic spines depicting morphological classification and changes in the cortex, CA1 stratum oriens and CA1 stratum radiatum of WT, *Tcf4*^*fl/+*^ and *Tcf4*^*fl/fl*^ animals. Color coding of the spine classes: red, mushroom; green, stubby; blue, thin. Scale bar, 2 μm. (B) Quantification of spine morphology after tamoxifen administration. In the cortex (left), *Tcf4*^*fl/+*^ and *Tcf4*^*fl/fl*^ dendrites display decreased numbers of stubby spines (n = 6; * p < 0.05) while only *Tcf4*^*fl/fl*^ neurons lose thin spines (* p < 0.05). In CA1 stratum oriens (middle) and stratum radiatum (right) the spine density of mushroom spines is affected in *Tcf4*^*fl/fl*^ neurons (n = 6; * p < 0.05). P values are based on ANOVA with Tukey’s multiple comparison test.

Since spine shape is intimately linked to function, we classed spines into three major morphological groups, mushroom, stubby and thin [30] (Fig 2A). In cortical layer V pyramidal cells, spine loss was carried mostly by a reduction in thin and stubby spines, while mushroom spines were affected to a lesser degree (Fig 2B). In hippocampal CA1 neurons, both on apical and basal dendrites, the overall reduction in spine was mostly caused by reductions in mushroom spines, which were the most abundant group in CA1 neurons (Fig 2B).

## Discussion

The number and shapes of dendritic spines are intimately linked to synaptic function. For instance, learning tasks in experimental animals lead to increased turnover and formation rates of dendritic spines. For instance, spines get formed and stabilized during motor learning [32] and repetitive motor learning caused newly formed spines to appear in clusters [33]. Neurodegenerative diseases, on the other hand are typically characterized by loss of dendritic spines [34], reflecting cognitive impairment and memory loss. Interestingly, however, reduced turnover of dendritic spines also causes cognitive impairment. This is the case in fragile X syndrome, where dendritic spines numbers are increased [35]. Furthermore, alterations in dendritic spines also occur in psychiatric diseases such as schizophrenia or autism-spectrum disorders, where alterations in spine density or turnover in the prefrontal cortex are thought to underlie cognitive and behavioral symptoms [19].

In the present study, we inquired whether Tcf4 plays a role in the maintenance of dendritic spines in adult animals, independently of its developmental functions. To achieve this, we generated a mouse line with inducible knockout of Tcf4 in a sparse subset of fluorescently labelled neurons. Our results suggest that heterozygous loss of Tcf4 suffices to substantially alter both spine density and morphology. These alterations may be the consequence of a disrupted negative feedback loop: Under normal conditions, elevated intracellular calcium, which may be the result of increased neuronal activity, inhibits the transcriptional activity of Tcf4 [36]. This may in turn dampen neuronal activity by removing or reshaping dendritic spines, in particular mushroom spines which, because of their large head surface, can generate stronger postsynaptic signals [23, 26]. Knockout of Tcf4 may thus precipitate these dampening effects even in the absence of increased neuronal activity.

The present results favor a postsynaptic, or cell-autonomous, role of Tcf4: In Slick-V mice, only a small fraction of neurons, both in the hippocampus and cerebral cortex, express cre (cf. Fig 1B). Therefore, the vast majority of presynaptic inputs into any given neuron are expected to be made up from processes of neurons not expressing cre recombinase, thus always expressing wildtype Tcf4. The genotype of the fluorescent neurons, in contrast, matches the induced alteration, i.e. wildtype, heterozygous loss of Tcf4 after tamoxifen administration (*Tcf4*^*fl/+*^), or homozygous loss (*Tcf4*^*fl/fl*^), with spine densities clearly correlating with these genotypes.

These results suggest that Tcf4 plays an important role in the control of synaptic plasticity, in adult animals, independent of the developmental function of Tcf4. Mouse models of Pitt-Hopkins syndrome, lacking functional Tcf4, typically show hyperactivity, reduced anxiety, and deficient spatial and associative learning [37, 38]. A reduced number of cortical synapses in humans was shown to correlate with decreased cognitive performance in the context of Alzheimer’s disease [39, 40]. Similarly, in various animal models, spine numbers and turnover have been shown to correlate with learning and memory and pathological alterations typically lead to decreased cognitive performance [24, 41]. Thus, based on our findings, we hypothesize that constitutive lack of functional Tcf4 causes cognitive and memory defects even in the absence of its neurodevelopmental effects. Thus, the clinical effects of loss of Tcf4 in Pitt-Hopkins syndrome may, at least in part, also be mediated by the synaptic function of Tcf4.
